# Hsa-miR-10a-5p downregulation in mutant UQCRB-expressing cells promotes the cholesterol biosynthesis pathway

**DOI:** 10.1038/s41598-018-30530-6

**Published:** 2018-08-17

**Authors:** Jeong Eun Kim, Ji Won Hong, Hannah S. Lee, Wankyu Kim, Jisun Lim, Yoon Shin Cho, Ho Jeong Kwon

**Affiliations:** 1 Chemical Genomics Global Research Lab., Department of Biotechnology, College of Life Science & Biotechnology, Yonsei University, Seoul, 120-749 Republic of Korea; 2 Department of Internal Medicine, Yonsei University College of Medicine, Seoul, 120-752 Republic of Korea; 3 Ewha Research Center for Systems Biology, Division of Molecular & Life Sciences, Ewha Womans University, Seoul, Republic of Korea; 4 Department of Biomedical Science, Hallym University, Chuncheon, Gangwon-do Republic of Korea

## Abstract

Ubiquinol cytochrome c reductase binding protein (UQCRB) is known to play crucial roles in the development of various types of diseases. However, the link between UQCRB and microRNAs remains unknown. In the present study, we performed microRNA sequencing of mutant UQCRB-expressing stable cell lines that exhibited pro-oncogenic activities caused by expression of the mutant UQCRB gene. Results showed that hsa-miR-10a-5p was significantly downregulated in the mutant UQCRB-expressing cell lines. Furthermore, mRNA sequencing and gene ontology analysis of differentially expressed genes (DEGs) revealed that the cholesterol biosynthesis pathway might be activation by mutant UQCRB expression. Moreover, inhibition of cholesterol synthesis in mutant UQCRB-expressing cells via treatment with the specific inhibitors suppressed the cell proliferation. Transfection with a hsa-miR-10a-5p mimic validated that lanosterol synthase (LSS) is a target of hsa-miR-10a-5p. In addition, hsa-miR-10a-5p was found to be downregulated in liver cancer cell lines overexpressing UQCRB. Taken together, our findings highlighted the potential use of hsa-miR-10a-5p as a biomarker for UQCRB related diseases.

## Introduction

Mitochondrial dysfunction has been implicated to play a key role in various diseases, such as metabolic diseases and cancer^1–5^. The electron transport complex (ETC) in the mitochondria consists of five complexes that involves in producing an electrochemical proton gradient for energy production by ATP synthesis^1^. The ubiquinol-cytochrome c reductase binding protein (UQCRB) is one of the subunits of mitochondrial complex III that plays a role in electron transport and maintenance of the mitochondrial complex III^6^. Identification of a target protein of terpestacin, an anti-angiogenic natural product, revealed a new role of UQCRB in regulation of mitochondrial ROS (mROS) generation and angiogenesis^7^. Moreover, many reports have implicated UQCRB variants in a number of diseases, including hepatocellular carcinoma^8^, ovarian cancer^9^, pancreatic ductal adenocarcinoma^10^, and colorectal cancer^11^.

In a recent case study, a Turkish female harboring a deletion in the gene encoding the UQCRB and isolated complex III defect presented hypoglycemia and lactic acidosis during a metabolic crisis in her babyhood; however, these conditions did not continue to her childhood^12^. Based on the above findings, our group generated mutant UQCRB-expressing stable cell lines, namely, MT1 and MT2, and investigated their angiogenic properties. The MT1 showed a higher expression level of mutant UQCRB protein than MT2 and both cell lines showed significantly faster cell growth and pro-angiogenic activities than those of control host human embryonic kidney cells 293 (HEK293). In addition, we demonstrated that treatment of these mutant UQCRB-expressing stable cell lines with UQCRB inhibitors significantly suppressed cell proliferation of the cells^13^.

MicroRNAs (miRNAs) are non-coding, single-stranded RNAs containing approximately 22 nucleotides. MicroRNAs are crucial regulators of numerous physiological and pathological processes^14,15^. Multiple studies have reported the use of miRNAs as biomarkers for specific diseases^16,17^. Furthermore, miRNAs have been implicated in mitochondrial function, metabolism, and metabolic disorders, such as cholesterol metabolism^18–20^.

However, the link between UQCRB and miRNAs remains to be mostly uncovered. In the present study, we performed microRNA and mRNA deep sequencing of mutant UQCRB-expressing stable cell lines with control host cell HEK293 to identify novel microRNA biomarkers for UQCRB related diseases.

## Results

### Identification of downregulated miRNAs in mutant UQCRB-expressing stable cell lines

Mutant UQCRB-expressing stable cell lines were subjected to miRNA sequencing to identify differentially expressed miRNAs that are specific to mutant UQCRB. The mutant UQCRB-expressing cell lines (MT1 and MT2) were previously generated based on a human case report expressing the mutant UQCRB gene. miRNA sequencing was conducted to compare the expression patterns of the generated mutant UQCRB-expressing cell lines with those of normal HEK293.

**Figure 1:**
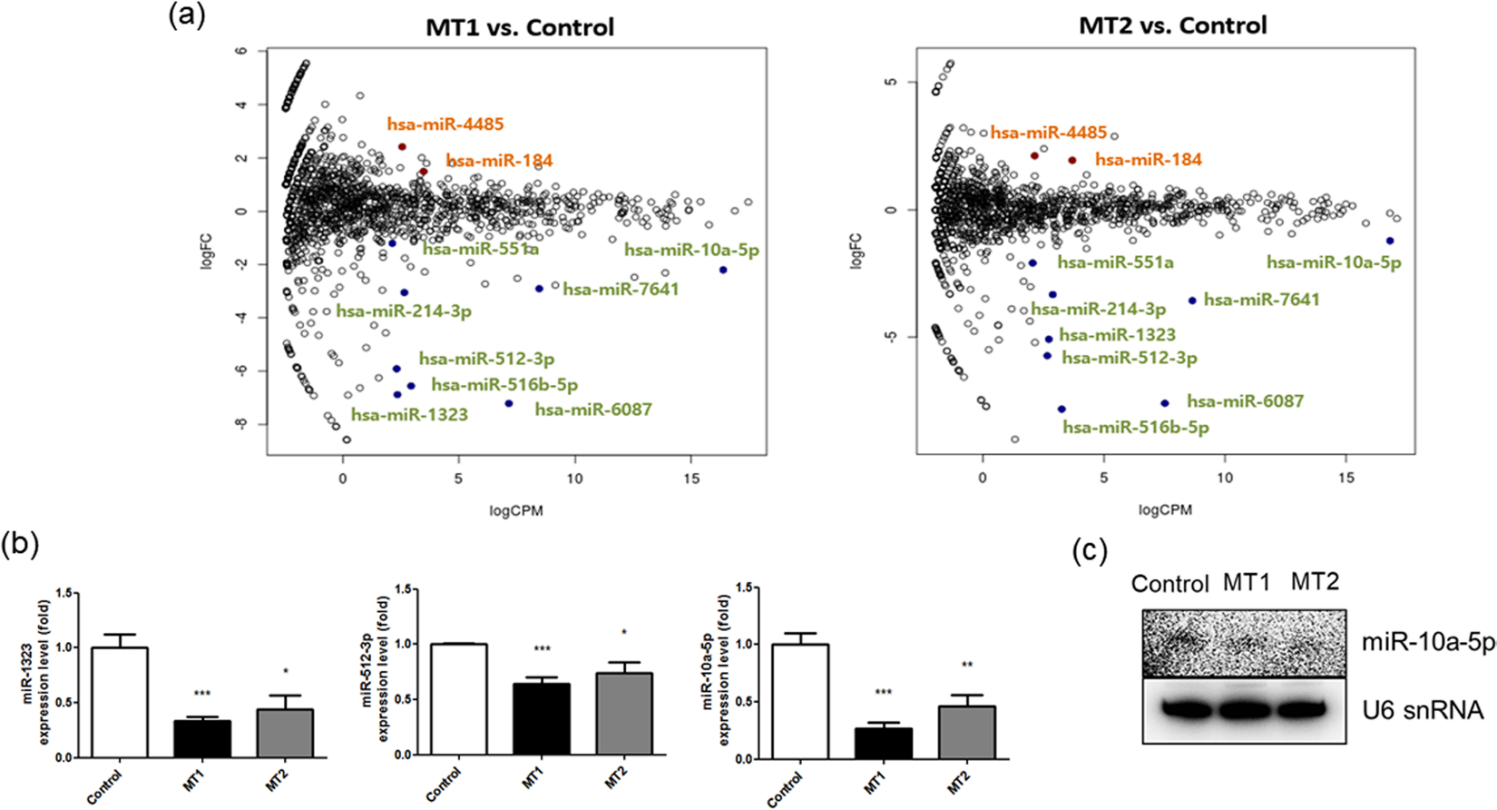
Hsa-miR-10a-5p was significantly downregulated in mutant UQCRB-expressing cell lines. (**a**) miRNA sequencing of mutant UQCRB-expressing cell lines and HEK293 cells identified key candidate miRNAs associated with mutant UQCRB. (**b**) qRT-PCR analysis validated the downregulation of three selected miRNAs in mutant UQCRB-expressing cell lines relative to HEK293. All data are presented as mean ± S.E.M. relative to the control. (*p < 0.05, **p < 0.01, ***p < 0.001). (**c**) miRNA expression levels of hsa-miR-10a-5p were evaluated via northern blotting. U6 snRNA was used as an internal control.

Our analysis identified more than 1,000 differentially expressed miRNAs in the mutant UQCRB-expressing cell lines, respectively. Twelve key candidate miRNAs that were differentially expressed between the mutant UQCRB and HEK293 cell lines were selected based on the following criteria: |log_2_FC| > 1, log_2_CPM > 2, and FDR < 0.15. Of these, nine miRNAs were downregulated (hsa-miR-6087, hsa-miR-1323, hsa-miR-516b, hsa-miR-512-3p, hsa-miR-214-3p, hsa-miR-7641, hsa-miR-10a-5p, hsa-miR-199b-3p and hsa-miR-551a), whereas three miRNAs were upregulated (hsa-miR-184, hsa-miR-1908-3p and hsa-miR-4485) compared to the control (Table 1). Quantitative RT-PCR was performed to further validate the miRNA sequencing results with seven down-regulated miRNAs and two up-regulated miRNAs. Suitable primers for hsa-miR-6087, miR-1908-3p, miR-199b-3p were unable to be designed so we didn’t validate these miRNAs expression levels in mutant UQCRB-expressing cells. Five out of the twelve miRNA candidates were validated to be down regulated in both mutant UQCRB-expressing cell lines. The five miRNAs included hsa-miR-1323, hsa-miR-214-3p, hsa-miR-512-3p, hsa-miR-10a-5p, and hsa-miR-551a. Hsa-miR-7641 was significantly downregulated in MT1 but not in MT2 and was therefore not considered as a mutant UQCRB-specific miRNA. In addition, hsa-miR-516b-5p was not downregulated in both mutant UQCRB-expressing cell lines, either (Supplementary Fig. 1a). qRT-PCR analysis did not show upregulated miRNAs (miR-184 and miR-4485) in both UQCRB mutant cell lines (Supplementary Fig. 1b). Out of the five validated miRNA candidates, three miRNAs, hsa-miR-10a-5p, hsa-miR-512-3p and hsa-miR-1323, were inverse correlation with mutant UQCRB-expressing stable cell lines suggesting that each miRNA were shown lower in MT1 cell than MT2 cell in both qPCR validation and informatics results (Fig. 1b and Table 1). However, validation using northern blot analysis showed that only hsa-miR-10a-5p was consistently downregulated (Fig. 1c). Therefore, we selected hsa-miR-10a-5p as the final candidate miRNA associated with UQCRB mutation.

**Table 1:** miRNAs were significantly up or downregulated in mutant UQCRB-expressing cells compared to the control.

miRNA(12)	MT1			MT2		
	log_2_FC	log_2_CPM	FDR	log_2_FC	log_2_CPM	FDR
Hsa-miR-6087	−7.21	7.14	2.72.E-32	−7.59	7.52	4.27.E-41
Hsa-miR-1323	−6.88	2.31	4.47.E-22	−5.08	2.73	1.07.E-16
Hsa-miR-516b-5p	−6.55	2.91	3.78.E-23	−7.82	3.26	1.89.E-25
Hsa-miR-512-3p	−5.91	2.27	2.16.E-19	−5.72	2.66	5.77.E-18
Hsa-miR-214-3p	−3.05	2.62	1.48.E-07	−3.32	2.89	8.75.E-10
Hsa-miR-7641	−2.9	8.45	1.40.E-07	−3.56	8.65	9.71.E-14
Hsa-miR-10a-5p	−2.2	16.38	1.35.E-04	−1.22	16.81	5.08.E-02
Hsa-miR-199b-3p	−1.76	2.65	7.79.E-03	−1.19	3.01	9.14.E-02
Hsa-miR-551a	−1.2	2.1	1.37.E-01	−2.09	2.1	4.48.E-04
Hsa-miR-184	1.49	3.48	6.00.E-02	1.94	3.7	3.32.E-04
Hsa-miR-1908-3p	1.79	4.74	1.30.E-02	1.37	4.29	2.57.E-02
Hsa-miR-4485	2.41	2.57	6.54.E-04	2.12	2.15	4.11.E-04

### Cholesterol synthesis pathway-related mRNAs were upregulated in mutant UQCRB-expressing cell lines

We next analyzed the differentially expressed mRNAs (DEGs) and related pathways to identify novel mutant UQCRB-associated phenotypes. mRNA sequencing was performed to compare gene expression patterns between HEK293 wild-type cells and mutant UQCRB-expressing cell lines (MT1 and MT2). For DEG analysis, candidate differentially expressed mRNAs were selected based on the criteria |fold change| > 1.5 and p-value < 0.01.

In addition, we performed gene set analysis to identify novel pathway. Results of gene set analysis showed significant upregulation (Benjamini <0.05) of eight different ontologies in both mutant UQCRB-expressing cell lines, which include sterol metabolic process, cholesterol metabolic process, steroid metabolic process, sterol biosynthetic process, steroid biosynthetic process (GO:0006694), terpenoid backbone biosynthesis, isoprenoid biosynthetic process, and cholesterol biosynthetic process. Additionally, the MT1 cell line showed upregulation in lipid biosynthetic process, steroid biosynthesis (hsa00100), MAPK signaling pathway, and biosynthesis of unsaturated fatty acids, whereas the MT2 cell line showed upregulation of mRNAs involved in isoprenoid metabolic process (Table 2). Most of significant gene ontologies were related with each other. Particularly, cholesterol synthesis pathway is most specific pathway that was finally selected to further explore novel biological roles of mutant UQCRB.

**Table 2:** mRNAs were significantly upregulated in mutant UQCRB-expressing cells compared to the control.

Term	Count	List Total	Pop Hits	Pop Total	Benjamini
● Mutant 1	Tool: DAVID, Cutoff: Benjamini <0.05				
GO:0016126~sterol biosynthetic process	10	118	35	13528	1.36.E-08
GO:0016125~sterol metabolic process	12	118	101	13528	6.10.E-07
GO:0006695~cholesterol biosynthetic process	8	118	26	13528	6.78.E-07
GO:0006694~steroid biosynthetic process	11	118	85	13528	8.45.E-07
GO:0008203~cholesterol metabolic process	11	118	92	13528	1.48.E-06
GO:0008610~lipid biosynthetic process	16	118	323	13528	2.31.E-05
GO:0008202~steroid metabolic process	13	118	202	13528	2.89.E-05
hsa00100:Steroid biosynthesis	6	55	17	5085	5.72.E-05
GO:0008299~isoprenoid biosynthetic process	5	118	20	13528	3.39.E-03
hsa00900:Terpenoid backbone biosynthesis	4	55	15	5085	2.12.E-02
hsa04010:MAPK signaling pathway	10	55	267	5085	3.92.E-02
hsa01040:Biosynthesis of unsaturated fatty acids	4	55	22	5085	4.48.E-02
● **Mutant 2**	**Tool: DAVID**, **Cutoff: Benjamini** <**0**.**05**				
GO:0016125~sterol metabolic process	666	15	1011101	13513528	5.64.E-06
GO:0008203~cholesterol metabolic process	6	15	92	13528	7.04.E-06
GO:0008202~steroid metabolic process	6	15	202	13528	1.20.E-04
GO:0016126~sterol biosynthetic process	4	15	35	13528	4.01.E-04
GO:0006694~steroid biosynthetic process	4	15	85	13528	4.68.E-03
hsa00900:Terpenoid backbone biosynthesis	3	11	15	5085	6.47.E-03
GO:0008299~isoprenoid biosynthetic process	3	15	20	13528	8.78.E-03
GO:0006695~cholesterol biosynthetic process	3	15	26	13528	1.28.E-02
GO:0006720~isoprenoid metabolic process	3	15	44	13528	3.20.E-02

**Figure 2:**
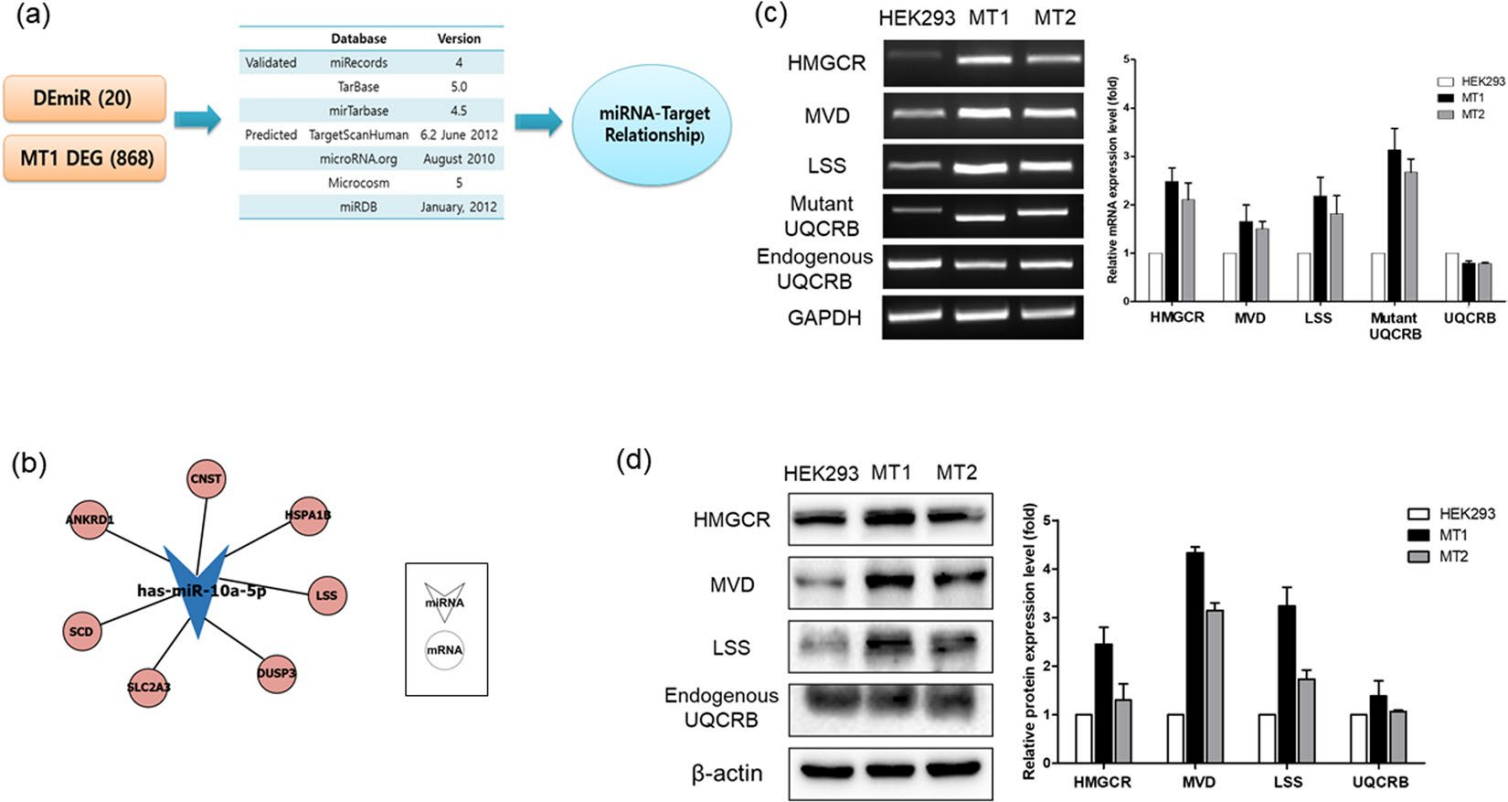
The potential relationship between differentially expressed miRNAs (DEmiRNAs) and differentially expressed genes (DEGs). (**a**) Scheme showing the integrated analysis workflow. Integration of 20 DE miRNAs and 868 DEGs in MT1 using seven databases. Of these, three databases contained validated miRNA targets, while the other databases contained miRNA target predictions. (**b**) Analysis of all miRNA-mRNA pairs identified putative associations of hsa-miR-10a-5p with seven mRNAs, including LSS. (**c**) RT-PCR was performed to evaluate the mRNA expression levels of HMG-CoA reductase (HMGCR), pyrophosphomevalonate decarboxylase (MVD), lanosterol synthase (LSS), mutant UQCRB, and endogenous UQCRB. Cholesterol synthesis enzymes were found to be upregulated in mutant cells. GAPDH was used as an internal control. (**d**) Western blot was performed to determine the protein levels of HMGCR, MVD, LSS and endogenous UQCRB. Cholesterol synthesis enzymes were found to be upregulated in mutant cells. β-actin was used as internal control.

We performed integrative analysis to identify hsa-miR-10a-5p targets in candidate differentially expressed mRNAs of MT1 cell using seven different databases. The first set of databases included miRecords (ver. 4), Tarbase (ver. 5), and mirTarbase (ver. 4.5), which contained information on the validated mRNA targets of miRNAs. We also used miRNA target prediction databases, namely, TargetScanHuman (ver. 6.2, June 2012), microRNA.org (August 2010), Microcosm (ver. 5), and miRDB (January 2012) (Fig. 2a).

Results showed that seven mRNA targets, namely, ANKRD1, SCD, CNST, SLC2A3, DUSP3, LSS, and HSPA1B had significantly high target prediction values for hsa-miR-10a-5p (Fig. 2b). The result showed a correlation between cholesterol synthesis and expression levels of the candidate miRNAs. Therefore, we selected lanosterol synthase (LSS) as a putative target mRNA of hsa-miR-10a-5p for further analysis.

**Figure 3:**
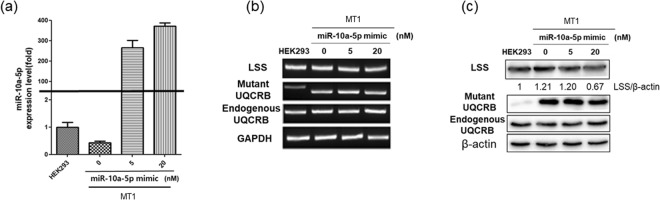
Transfection of mutant UQCRB-expressing cell line with a hsa-miR-10a-5p mimic. (**a**) qRT-PCR analysis showed that hsa-miR-10a-5p expression levels were upregulated in transfected mutant UQCRB-expressing cell line relative to non-transfected mutant UQCRB-expressing cells. (**b**) LSS mRNA expression levels were detected by RT-PCR. GAPDH was used as an internal control. (**c**) Western blotting showed that LSS protein levels were downregulated in MT1 cells. β-actin was used as internal control.

Next, we performed mimic transfection to validate that LSS could be the putative target of hsa-miR-10a-5p. Treatment with 5 nM and 20 nM of hsa-miR-10a-5p mimic miRNA was found to upregulate miRNA levels in a dose-dependent manner (Fig. 3a). LSS mRNA and protein levels were measured following transfection with 5 nM and 20 nM mimic. LSS expression levels were found to be upregulated in the MT1 cell line relative to those in the HEK293 cell line. However, treatment with 20 nM mimic downregulated both the mRNA and protein levels of LSS in a dose-dependent manner (Fig. 3b,c). Therefore, overexpression of hsa-miR-10a-5p downregulated the mRNA and protein levels of LSS. Collectively, the mutation in UQCRB might cause the downregulation of hsa-miR-10a-5p and hsa-miR-10a-5p upregulated LSS levels in the mutant UQCRB-expressing cell lines.

**Figure 4:**
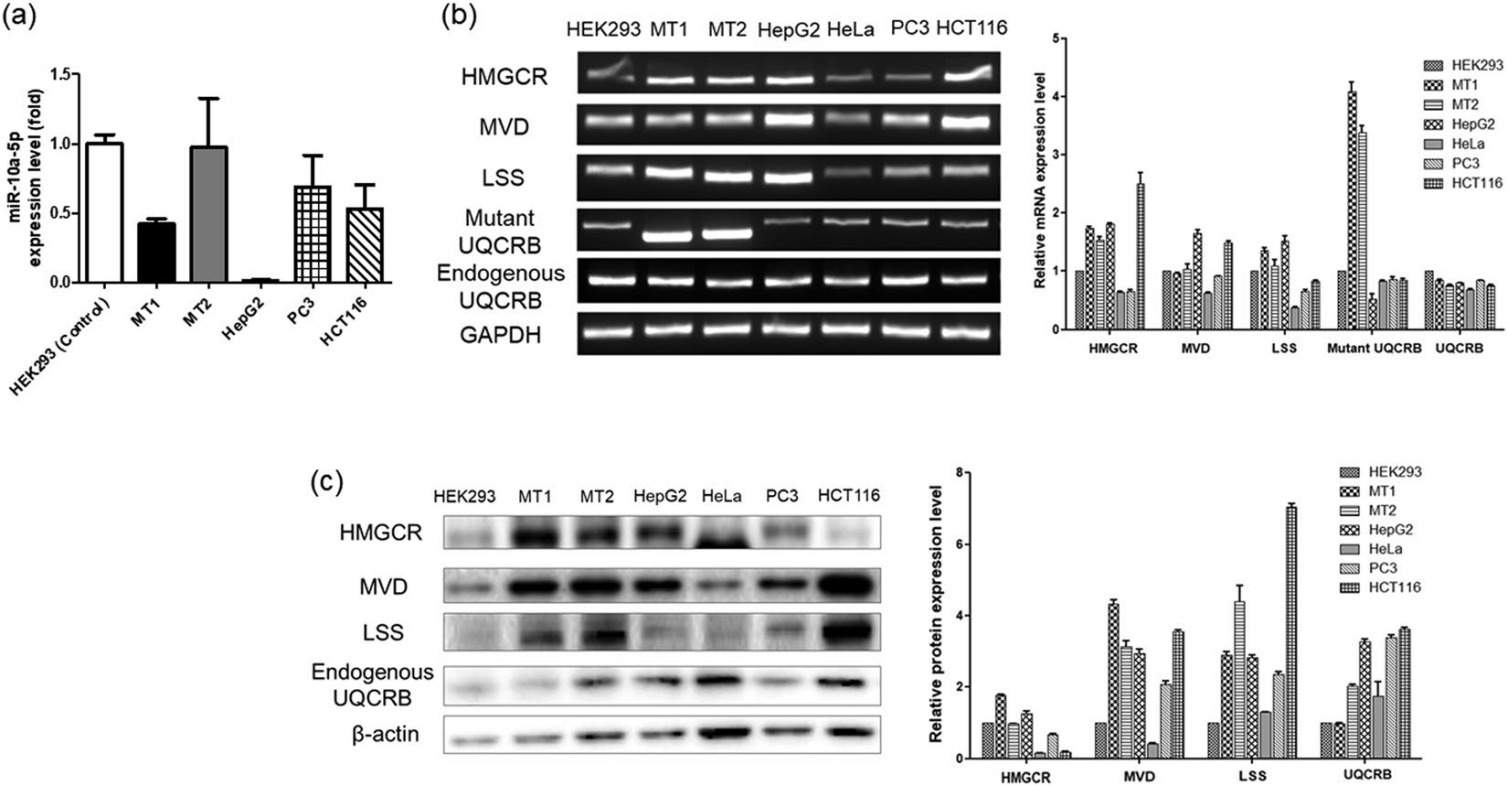
MicroRNA, mRNA, and protein expression levels of cholesterol synthesis enzymes in cell lines of mutant UQCRB-expressing cancer. (**a**) Hsa-miR-10a-5p expression levels were detected by qRT-PCR. Hsa-miR-10a-5p was found to be downregulated in HepG2, PC3 and HCT116 cells relative to HEK293 cells. All data are presented as mean ± S.E.M relative to control. (**b**) mRNA expression levels of three cholesterol synthesis-related enzymes determined via RT-PCR. Cholesterol synthesis related mRNAs, HMGCR, MVD, and LSS, were all upregulated in HepG2 cells relative to HEK293 cells. GAPDH was used as an internal control. (**c**) Protein expression levels of three cholesterol synthesis-related enzymes were determined via western blotting. HMGCR, MVD, and LSS levels were upregulated in HepG2 cells. β-actin was used as internal control. HepG2 cells showed similar expression patterns with those of mutant stable cell lines at the miRNA, mRNA, and protein level among the other cancer cells tested.

To further investigate the relationship between hsa-miR-10a-5p and UQCRB, we analyzed hsa-miR-10a-5p levels in cell lines of UQCRB expressing cancer, namely, HepG2 (human hepatocellular carcinoma), PC3 (human prostate cancer) and HCT116 cells (human colon cancer cell). Results of qRT-PCR analysis showed that hsa-miR-10a-5p levels were downregulated in all three cell lines tested with a variable degree than HEK293. In addition, hsa-miR-10a-5p level was also down-regulated in HeLa (human cervical cancer), which is also cell line of UQCRB expressing cancer (data is not shown). Notably, hsa-miR-10a-5p level was significantly downregulated in HepG2 was even lower than that in MT1 cells (Fig. 4a). Furthermore, the mRNA and protein levels of the examined cholesterol synthesis-related enzymes were further measured in four cell lines. mRNA levels of cholesterol synthesis-related enzymes were increased in HepG2 cells than HEK293 (Fig. 4b). Consistently, protein levels of the cholesterol synthesis-related enzymes were also increased in HepG2 cells than HEK293 (Fig. 4c).

**Figure 5:**
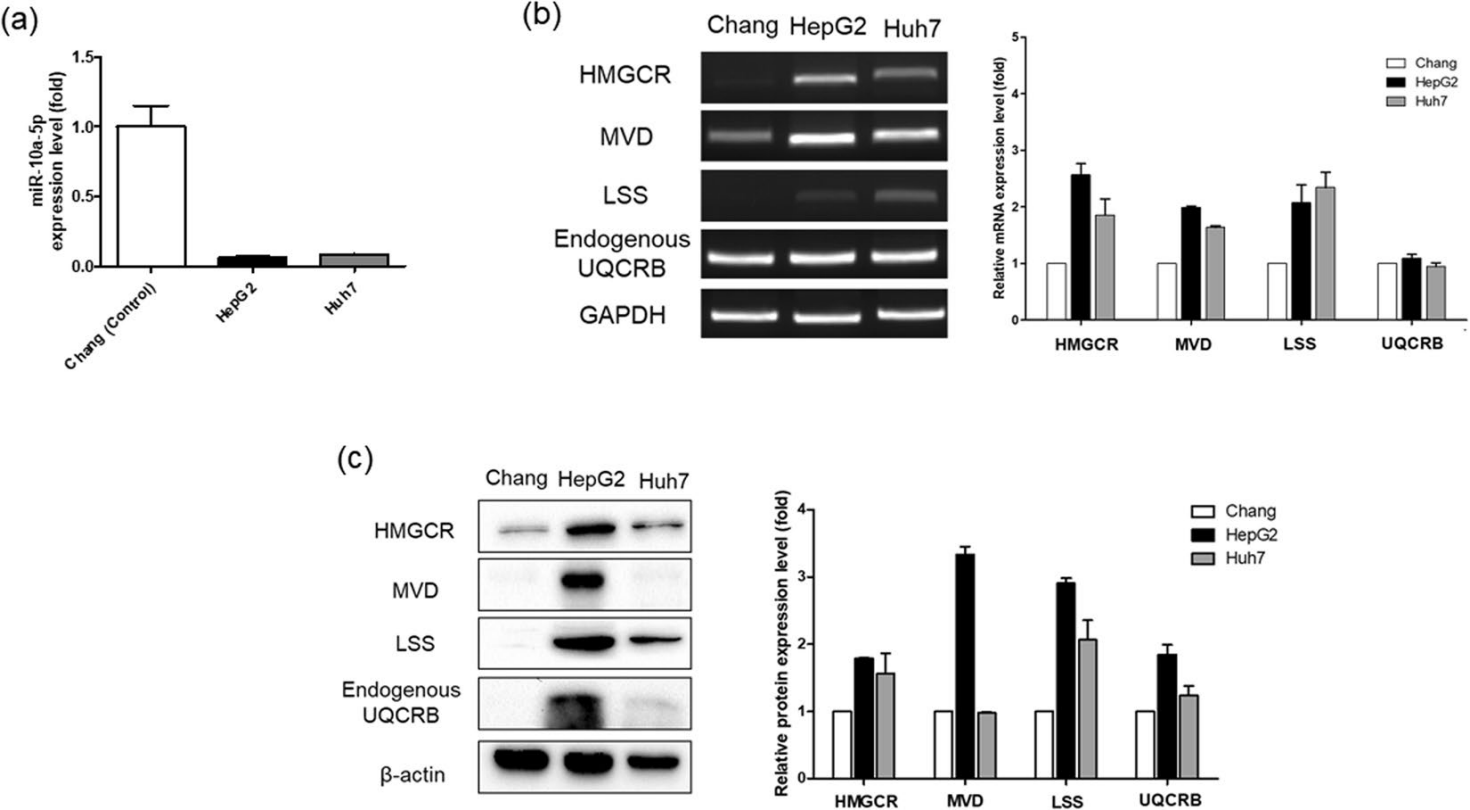
Comparison of expression level of microRNAs, cholesterol synthesis enzymes between liver cancer cells and normal liver cells. (**a**) Hsa-miR-10a-5p expression levels were detected via qRT-PCR. Hsa-miR-10a-5p was downregulated in HepG2 cells and Huh7 cells relative to Chang. All data are presented as mean ± S.E.M. relative to the control. (**b**) RT-PCR analysis showed that HMGCR, MVD, and LSS levels were upregulated in HepG2 and Huh7 compared to Chang cells. GAPDH was used as an internal control. (**c**) Protein levels of three cholesterol synthesis-related enzymes were detected via western blotting. Most of enzymes levels were upregulated in HepG2 in Huh7 cells. β-actin was used as internal control.

Given that hsa-miR-10a-5p levels were significantly downregulated in HepG2 cells than HEK293, we evaluated expression levels in a different liver cancer cell line, Huh7. Similar to the results from HepG2 cells, hsa-miR-10a-5p expression levels were significantly downregulated in Huh7 cells relative to those in Chang, a normal liver cell line (Fig. 5a). The mRNA levels of enzymes related to cholesterol synthesis were upregulated in HepG2 and Huh7 cells relative to those in Chang cells (Fig. 5b). Moreover, most of increased protein levels of enzymes related to cholesterol synthesis were also observed in HepG2 and Huh7 cells (Fig. 5c). These results implied that hsa-miR-10a-5p is significantly associated with UQCRB in liver cancer.

### Pro-proliferative activity of mutant UQCRB-expressing cell lines was regulated by cholesterol synthesis inhibitor

To further validate the relationship between mutant UQCRB and cholesterol synthesis, cell proliferation was evaluated after treatment with the following three different cholesterol synthesis inhibitors: Fatostatin, a sterol regulatory element-binding protein (SREBP) inhibitor^21^; Mevastatin, a HMG-CoA reductase (HMGCR) inhibitor; and YM-53601, a squalene synthase (FDFT1) inhibitor^22^. These inhibitors act on distinct points in the cholesterol synthesis pathway.

**Figure 6:**
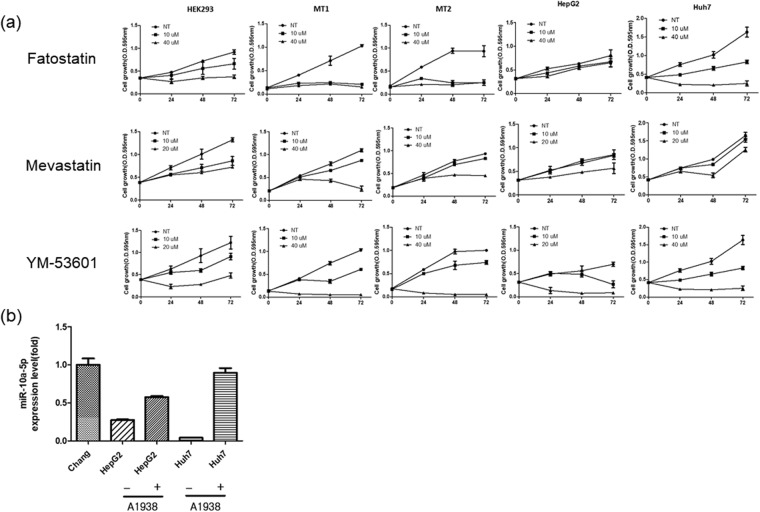
Regulation of cell growth by cholesterol synthesis inhibition. (**a**) HEK293, mutant UQCRB-expressing cells and liver cancer cell lines were treated with Fatostatin (10 µM, 40 µM), Mevastatin (10 µM, 20 µM), YM-53601 (10 µM, 20 µM) for 3 days. Cell growth was measured via the MTT colorimetric assay. (**b**) Effect of A1938, a UQCRB inhibitor, on hsa-miR-10a-5p expression levels in Chang and liver cancer cell lines. Cells were treated with A1938 (30 µM) for 36 h. All data are presented mean ± S.E.M.

MT1 and MT2 cells have higher proliferative activity than that of wild type HEK293 cells^13^. Notably, treatment of the mutant UQCRB-expressing cell lines with these inhibitors suppressed the cell growth in a dose-dependent manner as similar as HEK293. Similar results were obtained using liver cancer cell lines, as well. However, the efficacy of the cholesterol inhibitors varied depending on the liver cancer cell line used. Treatment with Fatostatin significantly inhibited cell proliferation in Huh7 cells. Mevastatin only slightly inhibited cell proliferation in both HepG2 and Huh7 cells. However, YM-53601 significantly inhibited cell proliferation in both HepG2 and Huh7 cells (Fig. 6a). These results suggested that the cholesterol synthesis pathway was activated in mutant UQCRB-expressing cells and that cell growth of mutant UQCRB stable cell lines might be dependent on cholesterol synthesis. Additionally, treatment with A1938, a UQCRB inhibitor, restored hsa-miR-10a-5p expression levels in liver cancer cell lines (Fig. 6b). These results demonstrate that the changes in cell proliferation were influenced by relationship of UQCRB and hsa-miR-10a-5p.

## Discussion

MicroRNAs have been reported as key regulators in various biological process. However, the link between UQCRB and miRNAs remains to be investigated. Here, we performed miRNA deep sequencing mutant UQCRB-expressing cell lines, which were used to investigate miRNA and the biological phenotypes that are associated with UQCRB mutant expression. The candidate miRNAs were selected using count per million (CPM) as the metric to reliable factors which even considered the basal expression level not only the difference of expression level in cell type.

Results of mRNA sequencing showed that cholesterol biosynthesis was activated in mutant UQCRB-expressing cell lines. Cholesterol synthesis is initiated by the mevalonate pathway, in which acetyl-CoA is a starting precursor and isopentenyl pyrophosphate (IPP) and dimethylallyl pyrophosphate (DMAPP) are the end products. The mevalonate pathway is crucial for *de novo* cholesterol biosynthesis and for the production of various intermediate metabolites that participate in other essential cellular processes^23^. For example, farnesyl pyrophosphate (FPP) and geranylgeranyl pyrophosphate (GGPP) are critical for post-translational modifications of GTPase proteins, including Ras and RhoA^24^. The mevalonate pathway has been demonstrated to be activated or inhibited in a number of different tumors and has been implicated in tumorigenesis. Furthermore, inhibitor of HMG-CoA reductase (HMGCR), a rate-limiting enzyme of mevalonate pathway, has been demonstrated to exert inhibitory effects on various types of cancers, including breast cancer^25–30^. Based on the above-mentioned findings, the oncogenic phenotype in mutant UQCRB-expressing cell lines (MT1 and MT2) could be induced at least in part by promoting cholesterol biosynthesis.

In the present study, we performed integrated analysis using established databases that provide validated microRNA-mRNA target pairs, as well as prediction databases that provide predicted microRNA-mRNA target pairs. Hsa-miR-10a-5p has been reported to regulate the metabolism in various cells including hypoxia related mechanism. Kinose Y *et al*. reported that hsa-10a-5p was down-regulated in CaOV3 and RMUG-S cells under hypoxia as compared to normoxia^31^. Consistent with this, our study also suggested that mutant UQCRB stable cell lines down-regulated hsa-miR-10a-5p that might lead to higher cell proliferation via promotion of cholesterol biosynthesis. On the contrary, however, Wu C *et al*. suggested that miR-10a was up regulated in hypoxia condition^32^ and Zhi F *et al*., suggested that hypoxia caused up-regulation miR-10a in rat liver^33^. Accordingly, the cause of down regulation of miR-10a-5p in mutant UQCRB stable cells and exact mechanism underlying this regulation needs to be investigated for addressing the complicated responses of miR-10a-5p upon cell types and physiological conditions in coming studies.

In the present study, the pro-proliferative activity of mutant UQCRB-expressing cell lines was regulated by cholesterol synthesis inhibitors. Accordingly, we thought that cholesterol synthesis pathway could play a role in pro-proliferative activity of these cell lines. As up-regulation of specific genes could influence to increase sensitivity or resistance to drug, further studies are also needed to explore the exact cause of these effects of inhibitors in respect with their sensitivity or toxicity to the cells.

Notably, the regulation of proliferative activity by cholesterol synthesis inhibitors in liver cancer cells was not so much effective compared to mutant UQCRB stable cell lines. This is probably because cholesterol synthesis may partially play a role in proliferative activity of mutant UQCRB cells. Because the other miRNAs also were down regulated in MT cells and these miRNAs could also play roles in proliferation through regulating other pathways of the cells, the effect of cholesterol synthesis inhibitors could be different in liver cancer cell lines.

In this study, we used mutant UQCRB stable cell lines to identify miRNAs and novel pathways related UQCRB. We consider that mutant UQCRB stable cell lines are similar with wild UQCRB expressing cells as UQCRB related pathways are similarly activated in the mutant UQCRB-expressing cells. According to previous studies of our group, supplement of UQCRB protein by transfection of wild type UQCRB gene^7^ and PTD-UQCRB treatment in cells and tissues^34^ increased mROS, stabilized HIF-1 and increased expression of VEGF. Consistently, mutant UQCRB-expressing cell lines showed similar properties as like these activities of wild UQCRB overexpression^13^. However, we are not able to exclude the fact that mutant UQCRB-expressing cell lines are not same with wild UQCRB expressing cell lines, exactly. To address this issue, detail comparative studies using wild and mutant UQCRB expressing cells are under investigation.

Collectively, our present study revealed that hsa-miR-10a-5p is associated with genes involved in the cholesterol synthesis pathway in mutant UQCRB-expressing cells. Moreover, LSS was validated as a hsa-miR-10a-5p target gene based on mimic transfection of mutant UQCRB-expressing cell lines. These results demonstrated that hsa-miR-10a-5p can be used as a biomarker for UQCRB related diseases.

## Materials and Methods

### Cell culture

Control (HEK293) cells, mutant UQCRB-expressing cell lines (MT1 and MT2), HeLa and HepG2 cells were grown in Dulbecco’s modified Eagle’s medium (DMEM; Invitrogen, Grand Island, NY) supplemented with 10% fetal bovine serum (FBS; Invitrogen) and 1% antibiotics (Invitrogen). All cells were incubated at 37 °C in a humidified incubator with 5% CO_2_ in air (pH 7.4). PC3 and HCT116 cells were grown in RPMI1640 (Invitrogen) under the same conditions described above. Mutant UQCRB stable cell lines were constantly provided with 1 mg/mL G418.

### RNA isolation

Cells were collected using TRIzol reagent (Invitrogen, Carlsbad, CA). Total RNA was extracted using PureLink^TM^ RNA isolation kit (Ambion) according to the manufacturer’s instructions.

### miRNA sequencing and miRNA expression

Isolated total RNAs were subjected to miRNA sequencing (Illumina HiSeq2000). Raw sequencing reads were provided by Macrogen (Macrogen Inc., Seoul, South Korea). Briefly, for miRNA-sequencing, small RNA sample preparation was performed according to the standard the Illumina protocols. The 5′ and 3′ adapters were sequentially ligated to small RNAs (18–30 bp) that were gel-purified from 5–10 µg of total RNA. Adapter-ligated small RNAs were reverse transcribed, amplified, and sequenced on a HiSeq2000 instrument (Illumina) according to the manufacturer’s instructions. Next, adapters were trimmed and mapped to the reference sequence using bowtie, an ultrafast, memory-efficient short read aligner designed to align large sets of short DNA sequences (reads) to large genomes^35^. Data were normalized via the quartile method using the edgeR package. Finally, candidate miRNAs were identified based on fold change (FC), count per million (CPM), and false discovery rate (FDR). The FDR was controlled by adjusting the p-values based on the Benjamini-Hochberg algorithm. The transcriptome analysis data of mutant UQCRB-expressing cells are available at Korean BioInformation Center (KBRS20171018–0000001~KBRS20171018–0000336).

### mRNA sequencing and mRNA expression quantitation

Isolated total RNAs were subjected to mRNA-sequencing (Illummina HiSeq2000). Raw reads were obtained from Macrogen (Macrogen Inc., Seoul, South Korea). cDNA library construction was performed using the TruSeq RNA library kit. Briefly, 1 µg of total RNA was subjected to polyA-selected RNA extraction, RNA fragmentation, random hexamer primed reverse transcription, and 100-nt paired-end sequencing on an Illumina HiSeq2000 instrument. The generated libraries were quantified via qPCR according to the qPCR Quantification Protocol Guide and validated using an Agilent Technologies 2100 Bioanalyzer. Next, the adapters were trimmed, and the resulting reads were mapped to the reference sequence using TopHat2. The reference genome sequence (hg19, Genome Reference Consortium GPCh37) and annotation data were downloaded from the UCSC website (http://genome.ucsc.edu). Read counts were analyzed, and differentially expressed mRNAs were identified based on fold change and p-value cutoffs using Cuffdiff.

### Quantitative RT-PCR (qRT-PCR)

RNA was reverse-transcribed using TaqMan MicroRNA reverse transcription kit (Applied Biosystems, Waltham, MA) and Taqman primers (Applied Biosystems). Real-time PCR was performed on a HT Fast Real Time PCR system (Applied Biosystems) using Taqman Fast Universal PCR master mix (Applied Biosystems) or on a LightCycler 96 system (Roche) with FastSTtart Essential DNA Probes Master (Roche) and TaqMan primers (Applied Biosystems) according to the manufacturer’s instructions. Data were analyzed based on the 2^−ΔΔCT^ method. Expression levels were normalized using the small RNA RNU48 as internal control.

### Reverse transcription (RT-PCR)

Isolated RNAs were reverse-transcribed using the following target-specific primers: wild-type UQCRB, forward: 5′-ATGTGAATTCATGGCTGGTAAGCAGGCC-3′ reverse: 5′ ATGCCTCGAGCTTCTTTGCCCATTCTTC-3′; mutant UQCRB, forward: 5′-ATGTGAATTCATGGCTGGTAAGCAGGCC-3′; reverse: 5′-CTC GAGGCCGTCCTCGTAGCAGCTGCAGCCGCACACCTCCACCACGTGGTTGCTGCGCTGGCCGTTCTTTTCTTTTCTTTCCCGAAT-3′; HMG-CoA reductase (HMGCR), forward: 5′-GTC ATT CCA GCC AAG GTT GT-3′; reverse: 5′-GGG ACC ACT TGC TTC CAT TA-3′; Mevalonate (diphospho) decarboxylase (MVD), forward: 5′-CTG CCT GAC TGC CTC AGC-3′; reverse: 5′-ACC TCT CCT GAC ACC TGG G-3′; lanosterol synthase (LSS), forward: 5′-GGC AGA CGT GGA CCT ACC T-3′; reverse: 5′-GAA AAG TGG GCC ACC ATA ATC-3′; GAPDH, forward: 5′-AACAGCGACACCCACTCCTC-3′; reverse: 5′-GGAGGGGAGATTCAGTGTGGT-3′). Expression levels were quantified using ImageJ 1.41o (NIH, USA) and Image LabTM software (Bio-rad).

### Western blot analysis

Cell lysates were analyzed via 10% and 12.5% sodium dodecyl sulfate polyacrylamide gel electrophoresis (SDS-PAGE) and subsequently transferred to polyvinylidenedifluoride membranes (Millipore, Billerica, MA) following standard methods. Blots were incubated at 4 °C overnight with the following primary antibodies: anti-UQCRB (Sigma-Aldrich, Saint Louis, MO), anti-HMGCR (Millipore), anti-MVD (Abcam, Cambridge, MA), anti-LSS (Abcam, Cambridge, MA), and β-actin (Abcam, Cambridge, MA). Immunolabeling was performed using Clarity Western ECL substrate (Bio-rad, Hercules, CA). Images were quantified using Image Lab^TM^ software (Bio-rad).

### miRNA mimic transfection

Mutant UQCRB-expressing cells were transfected with a miR-10a-5p mimic purchased from Ambion (ThermoFisher Scientific, Seoul, South Korea). Afterwards, 1.5 × 10^5^ cells were seeded onto six-well plates at 24 h prior to transfection. Transfection was performed using Lipofectamine 2000 (Invitrogen) reagent according to the manufacturer’s instructions. Next, cells were transfected with 5 and 20 nmol/L miRNA mimics after 48 h of incubation. Cells were then lysed in TRIzol reagent (Invitrogen).

### Cell proliferation assay

Cells were seeded onto 96-well plates and incubated for 24 to 72 h. Cell proliferation was measured using 3-(4,5-dimethylthiazol-2-yl)-2,5-diphenyltetrazolium bromide (MTT; Sigma-Aldrich, Saint Louis, MO) colorimetric assay. Cells were treated with Fatostatin (Sigma), Mevastatin (Sigma) and YM-53601 (Cayman chemicals, Ann Arbor, MI) at or above their reported IC_50_ values in cells. Liver cancer cells (HepG2 and Huh7, 1.5 × 10^5^ cells) were seeded onto 6-well plates and incubated for 36 h with of A1938 (30 µM), a UQCRB inhibitor. A1938 was dissolved in DMSO.

### Gene set analysis (GSA)

Forgene functional analysis of differentially expressed mRNAs (DEGs) was performed using the DAVID tool (http://david.abcc.ncifcrf.gov/)^36^. Data were filtered based on the cutoff value of Benjamini <0.05.

### Statistical analysis

Results were expressed as mean ± standard error (±S.E.M.). All statistical analyses were performed using GraphPad Prism (ver. 5.00 for Windows, GraphPad Software, San Diego, CA, www.graphpad.com). Student’s tests were performed to determine significant differences between the control and test groups. Statistically significant differences were considered at p-value < 0.05 (*indicates p < 0.05, **indicated p < 0.01, ***indicates p < 0.001).

## Electronic supplementary material

Supplementary Information
