# Supplementary material for: Hsa-miR-10a-5p downregulation in mutant UQCRB-expressing cells promotes the cholesterol biosynthesis pathway
Source: Sci Rep. 2018 Aug 17;8:12407. doi: 10.1038/s41598-018-30530-6 (PMC6098055; doi:10.1038/s41598-018-30530-6)

Supplement data

Hsa-miR-10a-5p downregulation in mutant UQCRB-expressing cells promotes the cholesterol biosynthesis pathway

Jeong Eun Kim[1], Ji Won Hong[1], Hannah S. Lee[1], Wankyu Kim[3], Jisun Lim[4], Yoon Shin Cho[4], and Ho Jeong Kwon[1,2,*]

[1]Chemical Genomics Global Research Lab., Department of Biotechnology, College of Life Science & Biotechnology, Yonsei University, Seoul 120-749, Republic of Korea

[2]Department of Internal Medicine, Yonsei University College of Medicine, Seoul 120-752, Republic of Korea

[3]Ewha Research Center for Systems Biology, Division of Molecular & Life Sciences, Ewha Womans University, Seoul, Republic of Korea

[4]Department of Biomedical Science, Hallym University, Chuncheon, Gangwon-do, Republic of Korea

Running title: miR-10a-5p as a biomarker of UQCRB mutant cells

To whom correspondence should be addressed: Ho Jeong Kwon, [1]Chemical Genomics Global Research Laboratory, Department of Biotechnology, College of Life Science & Biotechnology, Yonsei University, Seoul 120-749, Republic of Korea, Tel:82-2-2123-5883; Fax:82-2-362-7265; E-mail: kwonhj@yonsei.ac.kr

Supplementary Figure 1 (a) qRT-PCR analysis of downregulated miRNAs in both mutant UQCRB-expressing cell lines relative to HEK293. **(b)** qRT-PCR analysis of upregulated miRNAs in both mutant UQCRB-expressing cell lines relative to HEK293. All data are presented as mean ± S.E.M. relative to the control (* p < 0.05, ** p < 0.01, *** p < 0.001).

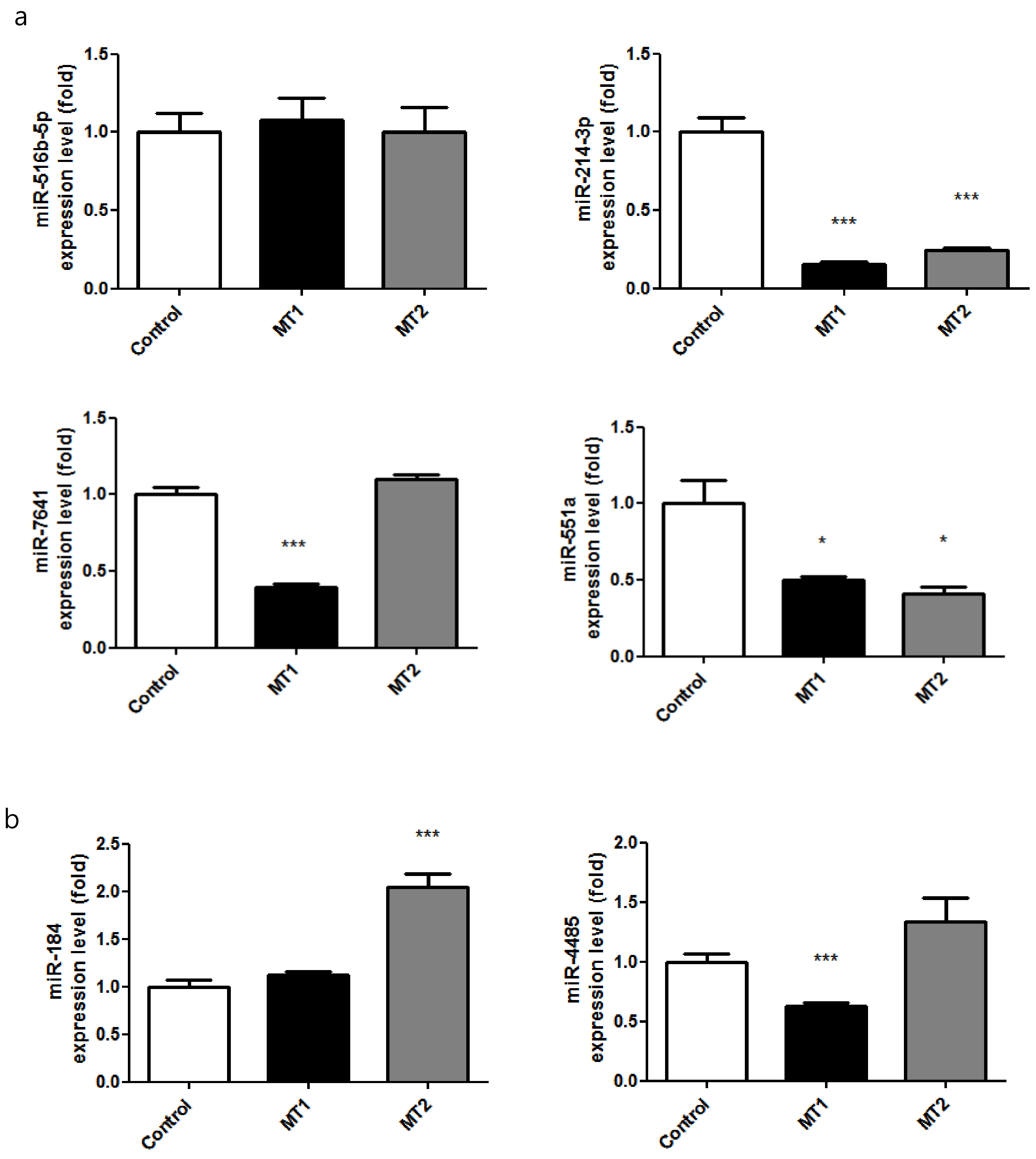

Supplement: Supplementary file 1 — Supplementary Information [file 41598_2018_30530_MOESM1_ESM.pdf]
